# Restricting the sizes of condensates

**DOI:** 10.7554/eLife.59663

**Published:** 2020-07-14

**Authors:** Furqan Dar, Rohit Pappu

**Affiliations:** 1 Department of Physics, Washington University in St. LouisSt. LouisUnited States; 2 Center for Science and Engineering of Living Systems, Washington University in St. LouisSt. LouisUnited States; 3 Department of Biomedical Engineering, Washington University in St. LouisSt. LouisUnited States

**Keywords:** multivalent proteins, phase separation, organelle sizes, metastable phase separation, metastable droplets, condensates, None

## Abstract

Computer simulations of model proteins with sticker-and-spacer architectures shed light on the formation of biomolecular condensates in cells.

**Related research article** Ranganathan S, Shakhnovich EI. 2020. Dynamic metastable long-living droplets formed by sticker-spacer proteins. *eLife* **9**:e56159. doi: 10.7554/eLife.56159

Many of the organelles found inside cells, including the nucleus and mitochondria, are enclosed within a membrane and have been closely studied for decades. However, there is growing interest in organelles that can form and dissolve reversibly because they are not surrounded by a membrane. In particular, the physics and chemistry of membraneless organelles – also known as biomolecular condensates – is the focus of much research (Banani et al., 2017; Shin and Brangwynne, 2017; Choi et al., 2020).

Biomolecular condensates form when a mixture of proteins, nucleic acids and solvents separate into a phase that is rich in proteins and nucleic acids, and a dilute phase that contains relatively few of these macromolecules. Basic thermodynamics suggests that this process of 'phase separation' should result in a single large condensate that co-exists with a dilute phase because the energy needed to maintain the interface between a single large condensate and a dilute phase is lower than the interfacial energy for a system of smaller condensates. A process known as Ostwald ripening establishes this equilibrium by allowing a single large condensate to incorporate smaller ones (Lifshitz and Slyozov, 1961).

Systems containing a single large condensate, as predicted by basic thermodynamics, have been observed in in vitro studies (Elbaum-Garfinkle et al., 2015). However, there have also been reports of living cells that contain multiple condensates that do not grow beyond a certain size (Brangwynne et al., 2009; Berry et al., 2018). The form of phase separation that yields multiple droplets or condensates – a process known as emulsification – is thought to arise from the active production and degradation of macromolecules (Wurtz and Lee, 2018). However, there have also been reports of emulsification happening in the absence of these active mechanisms. How can one explain emulsification when such processes are not at work?

Now, in eLife, Srivastav Ranganathan and Eugene Shakhnovich from Harvard University report the results of simulations modelling the phase behavior of model polymers made up of multiple 'stickers' and 'spacers' that help to answer this question (Ranganathan and Shakhnovich, 2020; Figure 1A). These simulations show that the sizes of condensates are determined by two timescales: the time it takes for macromolecules to come into contact via diffusion; and the time it takes to form and break physical bonds between pairs of ‘stickers’ (Figure 1A,B).

**Figure 1.**
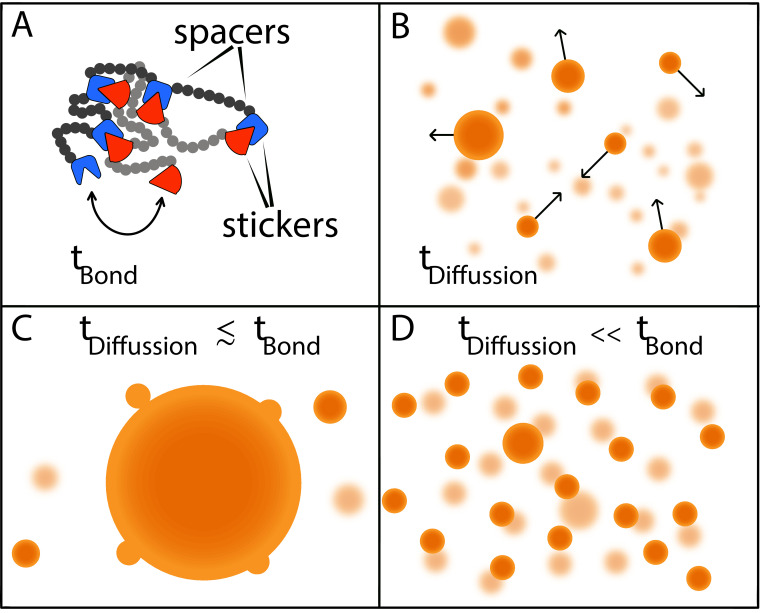
Two timescales determine the size of condensates. (**A**) Models in which macromolecules are composed of stickers and spacers can be used to predict the phase behavior of proteins (Choi et al., 2019). This schematic shows the interactions between two such macromolecules, with the stickers in one macromolecule (red shapes) forming bonds (reversible, non-covalent crosslinks) with the stickers in the other macromolecule (blue shapes); the spacers are shown as grey and black circles. Bonds between the stickers are made and broken on a time scale of *t_bond_*. (**B**) Free macromolecules (small orange spheres) diffuse and collide on a timescale of t_difffusion_, sometimes sticking together to form condensates (large orange spheres). (**C**) When these two timescales are roughly equal, a phenomenon known as Ostwald ripening leads to the formation of a dominant condensate that continues to grow by absorbing smaller condensates. (**D**) Ranganathan and Shakhnovich predict that when the timescale for diffusion is much faster than the timescale for making and breaking bonds, condensates cannot grow beyond a certain size, which results in a large number of small- and medium-sized condensates.

If these two timescales are similar to one another, larger condensates will consume smaller condensates until there is just one dominant condensate (Figure 1C). However, if the timescale for diffusion is orders of magnitude faster than the timescale for bond formation, as is more often the case, most of the inter-sticker bonds will form among molecules that are part of smaller condensates. Moreover, new molecules will not be able to join the condensate because most sticker regions will already be tied up in existing connections. In the absence of stickers to bind to, molecules that are not already in the condensate will diffuse away. As a result, while it is relatively easy to grow a condensate up to a certain size, the lack of available molecules to form bonds with limits further growth, resulting in a roughly homogeneous distribution of smaller condensates (Figure 1D).

There has been much debate over how emulsification arises in cells. In addition to active processes controlling the size of condensates, another possibility is that some cellular components act as surfactants to decrease the energy of the interface between condensates and the solvent (Cuylen et al., 2016). Ranganathan and Shakhnovich now offer a third possible explanation. A fourth possibility is that proteins with block copolymeric architectures (a chain with blocks of two or more distinct monomers) form condensates via micellization (Ruff et al., 2015).

Biology seems to find a way to leverage all aspects of physically feasible scenarios in order to achieve desired outcomes. This is clearly the case with regards to the size distribution and apparent emulsification of condensates. However, it remains unclear how these different modes of emulsification interact with one another and to what extent each of these modes is used by different cell types. Theory and computations have offered elegant, testable predictions that have paved the way for designing experiments that can answer these questions.
